# Systems Pharmacology and Multi-Omics Elucidation of *Irpex lacteus* Polysaccharides in the Treatment of Lupus Nephritis Through PI3K/AKT/NF-κB Pathway Inhibition

**DOI:** 10.3390/ph18111619

**Published:** 2025-10-27

**Authors:** Guoxin Ji, Zhuangzhuang Yao, Yuetong Zhao, Cuicui Li, Bo Yang, Zhimeng Li, Mingfang Kuang, He Wang, Xian Wu, Huiyang Yuan, Yue Deng, Shumin Wang, Huan Wang

**Affiliations:** 1 College of Pharmacy, Changchun University of Chinese Medicine, Changchun 130117, China; 17843099892@163.com (G.J.);; 2 College of Traditional Chinese Medicine, Changchun University of Chinese Medicine, Changchun 130117, China; 3 College of Integrated Chinese and Western Medicine, Changchun University of Chinese Medicine, Changchun 130117, China; 4 Ginseng Scientific Research Institute, Changchun University of Chinese Medicine, Changchun 130117, China

**Keywords:** *Irpex lacteus* polysaccharides, systemic lupus erythematosus, lupus nephritis, transcriptomics, metabolomics, PI3K/AKT/NF-κB signaling pathway

## Abstract

**Background:** Lupus nephritis (LN), a severe complication of systemic lupus erythematosus (SLE), necessitates effective therapeutic strategies. Polysaccharides derived from *Irpex lacteus* have demonstrated beneficial biological activities in MRL/lpr mice; however, their precise mechanisms of intervention in LN require further elucidation. **Methods:** MRL/lpr mice were administered low-dose and high-dose *Irpex lacteus* polysaccharide (PCP) continuously for 8 weeks. The therapeutic efficacy of PCP was systematically assessed by measuring autoantibody levels, inflammatory cytokine expression, and renal function markers. The underlying pharmacological mechanisms were investigated through integrated transcriptomics and metabolomics analyses. **Results:** PCP treatment significantly improved renal function in MRL/lpr mice, normalizing serum levels of anti-nuclear antibodies (ANA), anti-double-stranded DNA antibodies (anti-dsDNA), anti-Sm antibody (Sm), creatinine (Cr), blood urea nitrogen (BUN), interleukin-6 (IL-6), tumor necrosis factor-alpha (TNF-α), and proteinuria. Integrated transcriptomic and metabolomic analyses revealed that the therapeutic action of PCP involves modulation of the PI3K/AKT/NF-κB pathway. This inhibition was further confirmed by Western blot analysis. **Conclusions:** PCP exerts renal protective effects in MRL/lpr mice by mitigating inflammation, modulating immune responses, and preserving renal function. The combined application of transcriptomics, metabolomics, and Western blotting elucidates that this protection is mediated through inhibition of the PI3K/AKT/NF-κB signaling pathway.

## 1. Introduction

Systemic Lupus Erythematosus (SLE) is a chronic autoimmune disease affecting multiple systems, with its pathogenesis centered on the aberrant activation of autoreactive B cells and the excessive production of pathogenic autoantibodies, such as anti-nuclear antibodies (ANA) and anti-double-stranded DNA antibodies (anti-dsDNA) [1]. These antibodies cause widespread tissue damage by forming and depositing circulating immune complexes in target organs, such as the skin, joints, and kidneys, triggering complement activation and inflammatory cell infiltration [2]. Notably, lupus nephritis (LN), the most common and severe complication of SLE, affects over 50% of patients and is a leading cause of end-stage renal disease and mortality [3,4]. Clinically, the treatment of LN primarily involves regimens combining immunosuppressants with glucocorticoids, aiming to suppress the abnormal immune response against self-antigens. However, current therapies often fail to achieve complete disease remission [5]. Additionally, the long-term and high-dose use of these medications frequently leads to significant toxic side effects, negatively impacting patients’ survival and quality of life [6]. Therefore, the development of novel therapeutic strategies, designed to more effectively modulate immune balance and control inflammatory responses, holds critical importance for improving the prognosis of LN.

In the field of systems biology, metabolomics and transcriptomics serve as complementary key technologies, jointly forming the foundation for exploring the complex regulatory networks of biological systems [7]. Metabolomics captures the holistic landscape of small-molecule metabolites (such as sugars, amino acids, and lipids) within an organism, directly reflecting the phenotypic characteristics and immediate physiological state of the body [8]. Transcriptomics, on the other hand, employs high-throughput sequencing to map the expression profiles of all RNA transcripts (including mRNA and non-coding RNA) under specific conditions, revealing the fine-tuned regulatory processes of gene expression [9]. Although these two omics approaches have achieved remarkable success in their respective fields, their interplay in the context of LN, a complex disease, remains largely unexplored. Therefore, conducting an integrated analysis of transcriptomics and metabolomics holds great potential to unlock the complexity of LN and discover potential therapeutic targets or biomarkers.

The PI3K/AKT/NF-κB signaling pathway serves as a central hub connecting the immune-inflammatory response with renal cell injury, playing a crucial role in the pathogenesis of LN [10]. The aberrant activation of this pathway initially promotes the survival and differentiation of autoreactive B cells, leading to the massive production of pathogenic autoantibodies. These antibodies form immune complexes that deposit in the kidney and activate complement, constituting the initiating event in LN pathogenesis [11]. Subsequently, the pathway activates IκB kinase, which mediates the degradation of the NF-κB inhibitor, thereby liberating the core transcription factor NF-κB to initiate the transcription of pro-inflammatory factors and chemokines. This creates an “inflammatory storm” that recruits and activates more inflammatory cells to infiltrate the kidney, directly causing tissue damage [12]. Notably, AKT can directly phosphorylate the p65 subunit of NF-κB, and their synergistic interaction forms a positive feedback loop that sustains and amplifies the inflammatory response [13]. Moreover, the activated pathway also targets intrinsic renal cells: in glomerular mesangial cells, it promotes proliferation and extracellular matrix deposition, accelerating glomerulosclerosis; in renal tubular epithelial cells, it induces apoptosis and epithelial–mesenchymal transition. Together, these effects collectively drive the process of renal fibrosis, which serves as the key pathological basis for the progression of LN to end-stage renal disease [14].

*Irpex lacteus*, a white-rot basidiomycete, is widely used in environmental remediation for its robust lignin-degrading capabilities, particularly in the degradation of recalcitrant organic pollutants in contaminated soils and the decolorization of synthetic dyes. In recent years, its medicinal and edible value has garnered increasing attention, especially its key bioactive component—*Irpex lacteus* polysaccharides (PCP), which have been demonstrated to possess multiple biological activities, including anti-fatigue, anti-inflammatory, and immunomodulatory effects. Research indicates that PCP inhibits the proliferation of mesangial cells, providing a new clinical basis for the prevention and treatment of mesangial proliferative diseases and glomerulonephritis [15]. Furthermore, PCP has shown significant therapeutic effects in models of membranous nephropathy [16]. Notably, beyond its known efficacy in chronic glomerulonephritis, PCP also exhibits remarkable therapeutic potential in the treatment of LN, opening new avenues for its application in kidney diseases.

Addressing the treatment challenges of LN, this study is dedicated to systematically evaluating the therapeutic effects of PCP in the MRL/lpr mouse model, with a focus on monitoring its improvement on cytokine levels and renal pathological damage. Furthermore, the analysis of PCP’s regulatory patterns on transcriptomics and metabolomics aims to uncover its underlying mechanisms in treating LN, while also expanding its potential for development into functional foods and pharmaceuticals.

## 2. Results

### 2.1. PCP Physicochemical Characteristics

Table 1 summarizes the contents of polysaccharide, soluble glycoprotein, and sulfate within PCP. The polysaccharide content was the highest at 696.86 ± 6.54 mg/g, followed by sulfate radical at 94.40 ± 1.36 mg/g, and soluble protein at 11.51 ± 0.08 mg/g.

**Figure 1:**
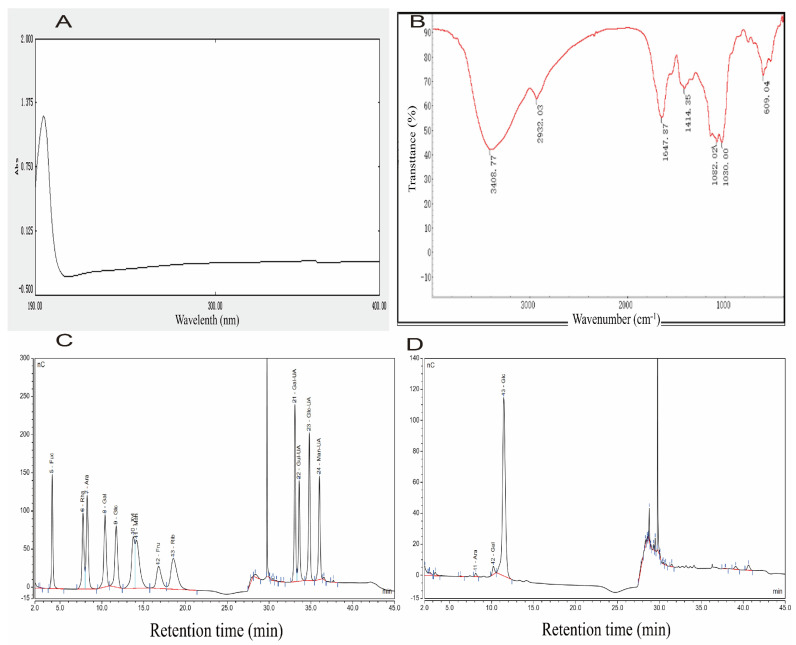
Spectral analysis of the PCP. (**A**) UV spectrum of PCP. (**B**) IR spectrum of PCP. (**C**,**D**) Monosaccharide composition of PCP.

To further accurately characterize PCP, ultraviolet (UV) and Infrared (IR) absorption spectroscopy were employed. The UV absorption spectrum exhibited a distinct peak at 195 nm (Figure 1A), which is characteristic of polysaccharides. Notably, no significant absorption peaks were observed at 260 nm and 280 nm, suggesting the presence of negligible quantities of nucleic acids and proteins in the sample. These findings indicate that the tested polysaccharide was extracted with high purity.

The IR spectrum of the sample provided further insights into its molecular structure (Figure 1B). The absorption band observed in the range of 3600–3200 cm^−1^ corresponds to the stretching vibration of -OH groups, which is a characteristic peak of carbohydrates. Specifically, the absorption peak at 3408.77 cm^−1^ is attributed to the stretching vibration of O-H, a hallmark of carbohydrates. Additionally, the absorption peak at 2932.03 cm^−1^ is indicative of the stretching vibration of C-H bonds, while the peak at 1082.02 cm^−1^ is associated with the stretching vibration of C-O bonds.

Analysis of monosaccharide composition using 13 monosaccharides, which are fucose (Fuc), rhamnose (Rha), arabinose (Ara), galactose (Gal), glucose (Glc), xylose (Xyl), mannose (Man), fructose (Fru), ribose (Rib), galacturonic acid (Gal-UA), glucuronic acid (Glc-UA), mannuronic acid (Man-UA), and guluronic acid (Gul-UA) as monosaccharide standards. As shown in Figure 1C,D, PCP is composed of Ara, Gal, and Glc, with a molar ratio of Ara: Gal: Glc of 1:2.08:64.3.

### 2.2. PCP Ameliorates Renal Function and Alleviates Kidney Injury by Modulating Autoantibody Levels and Inflammatory Cytokines in MRL/Lpr Mice

**Figure 2:**
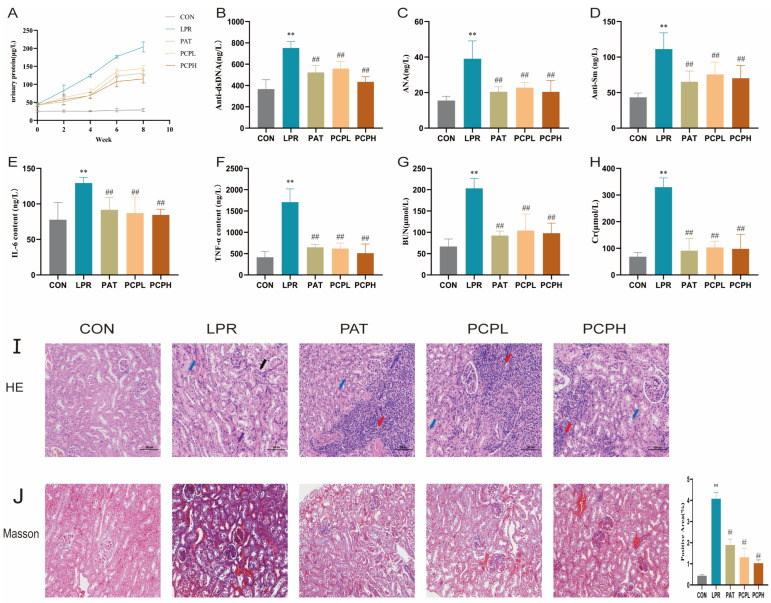
Comprehensive analysis of serum autoantibody levels, inflammatory cytokine levels, renal function indicators, and renal tissue histopathology. (**A**) UP. (**B**) ds-DNA. (**C**) ANA. (**D**) Sm. (**E**) IL-6. (**F**) TNF-α. (**G**) BUN. (**H**) Cr. Data were presented as mean ± SD (*n* = 6). Significant differences were indicated as ** *p* < 0.01 vs. CON. ^##^ *p* < 0.01 vs. LPR. (**I**) Renal H&E staining results. (**J**) Renal Masson’s trichrome staining results and the proportion of positive areas.

As shown in Figure 2A, the proteinuria level in the control (CON) group did not increase over time. In the model (LPR) group, proteinuria levels increased gradually over time, whereas positive control (PAT) and PCP treatment reduced proteinuria levels. As shown in Figure 2B–D, compared with the CON group, the levels of anti-dsDNA, ANA, and anti-Sm antibody (Sm) antibodies were significantly elevated in the LPR group (*p* < 0.01). The levels of anti-dsDNA, ANA, and Sm antibodies in the LPR group were 751.98 ng/L, 39.06 ng/L, and 111.25 ng/L, respectively. Compared to the LPR group, the levels of anti-dsDNA, ANA, and Sm antibodies in the PAT group recovered to 523.02 ng/L (*p* < 0.01), 20.49 ng/L (*p* < 0.01), and 65.30 ng/L (*p* < 0.01), respectively; in the low-dose PCP (PCPL) group, they recovered to 558.73 ng/L (*p* < 0.05), 22.77 ng/L (*p* < 0.01), and 75.54 ng/L (*p* < 0.01), respectively; and in the high-dose PCP (PCPH) group, they recovered to 434.52 ng/L (*p* < 0.01), 20.41 ng/L (*p* < 0.01), and 70.30 ng/L (*p* < 0.01), respectively. As shown in Figure 2E,F, compared with the CON group, the levels of tumor necrosis factor-alpha (TNF-α) and interleukin-6 (IL-6) were significantly increased in the LPR group (*p* < 0.01), and both the PCPL and PCPH groups were able to improve the levels of these inflammatory factors (*p* < 0.01). As shown in Figure 2G,H, the levels of creatinine (Cr) and blood urea nitrogen (BUN) in the LPR group were significantly higher than those in the CON group (*p* < 0.01). Both the PCPL and PCPH groups significantly reduced the levels of Cr and BUN (*p* < 0.01). Concurrently, the PAT group also showed improvement in all the aforementioned indicators.

Histological examination by Hematoxylin and Eosin (H & E) staining, as shown in Figure 2I, revealed that in the CON group, the renal tissue exhibited normal glomeruli with uniform cellularity and matrix. The tubular epithelial cells were round and plump, with regularly arranged brush borders, and no obvious abnormalities were observed. The renal interstitium, located between the urinary tubules, showed no significant hyperplasia, and no prominent inflammatory cell infiltration was detected. In the LPR group, the glomeruli in the renal tissue showed uniform cellularity and matrix. However, numerous tubular epithelial cells exhibited hydropic degeneration (blue arrows), characterized by loose and lightly stained cytoplasm. Occasionally, tubular epithelial cell necrosis (purple arrows) was observed, marked by pyknosis of the nucleus, increased eosinophilia of the cytoplasm, obscured luminal structure, and occasionally eosinophilic material within the tubules (black arrows). In the PAT group, the renal tissue displayed normal glomeruli with uniform cellularity and matrix. Numerous tubular epithelial cells showed hydropic degeneration (blue arrows), nuclear pyknosis, and increased cytoplasmic eosinophilia. A few focal lymphocytic infiltrates around blood vessels were observed in the interstitium (red arrows). In the PCPL group, the renal tissue showed normal glomeruli with uniform cellularity and matrix. Numerous tubular epithelial cells exhibited hydropic degeneration (blue arrows), with loose and lightly stained cytoplasm. Several focal lymphocytic infiltrates were observed in the interstitium (red arrows). In the PCPH group, the renal tissue presented normal glomeruli with uniform cellularity and matrix. Numerous tubular epithelial cells showed hydropic degeneration (blue arrows), with loose and lightly stained cytoplasm. Occasional small focal lymphocytic infiltrates were noted in the interstitium (red arrows).

Masson’s trichrome staining results, shown in Figure 2J, indicated that compared to the CON group, the proportion of positive areas in the LPR group significantly increased, reaching 4.08% (*p* < 0.01). The positive area proportion in the PAT group was 1.89% (*p* < 0.01), in the PCPL group it was 1.31% (*p* < 0.01), and it was further reduced to 1.03% in the PCPH group (*p* < 0.01).

### 2.3. Transcriptome Analysis Results

**Figure 3:**
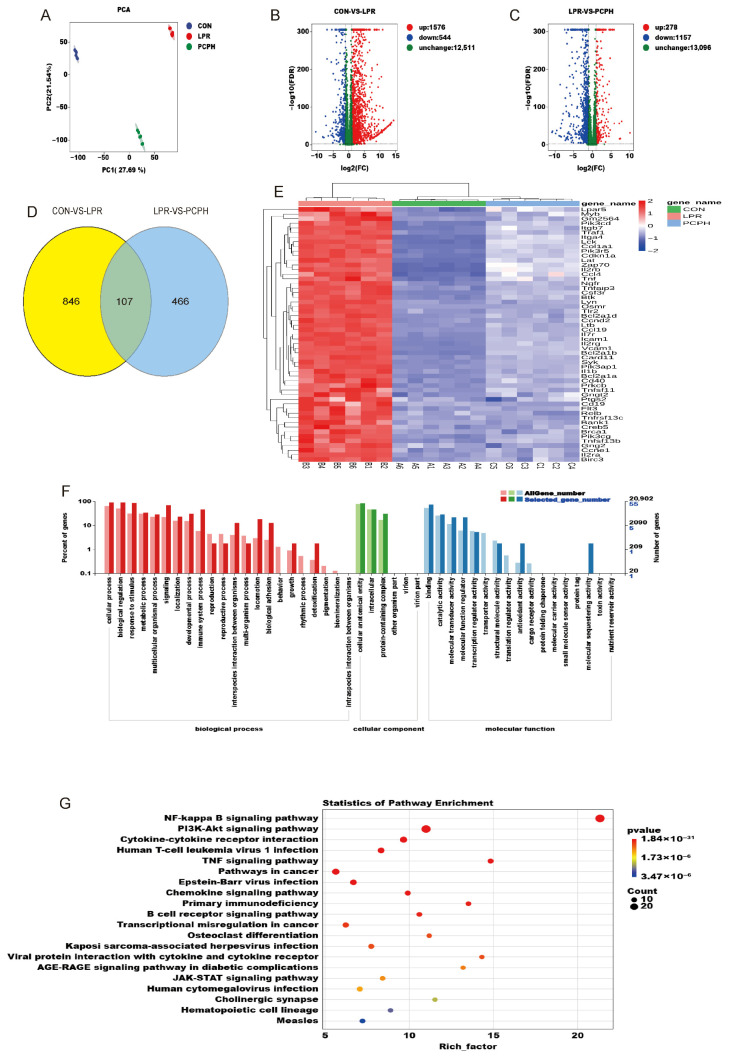
PCPH regulates the expression of LN-related genes (*n* = 6). (**A**) PCA. (**B**) Volcano plot of DEGs between the CON and LPR groups. (**C**) Volcano plot of DEGs between the LPR and PCPH groups. (**D**) Venn diagram of overlapping DEGs between the CON-vs.-LPR and LPR-vs.-PCPH comparison groups. (**E**) Hierarchical clustering heatmap of DEGs. (**F**) Results of GO analysis for DEGs. (**G**) Results of KEGG analysis for DEGs.

RNA-seq analysis was performed to compare the transcriptomes of the CON, LPR, and PCPH groups, completing the eukaryotic reference-based transcriptome analysis of 18 samples, yielding a total of 153.14 Gb of Clean Data. Each sample’s Clean Data reached 5.49 Gb, with a Q30 base percentage of 93.66% or higher. Subsequently, the Clean Reads of each sample were aligned to the designated reference genome, with mapping efficiencies ranging from 95.93% to 97.31%, indicating high-quality transcriptome sequencing data. The principal component analysis (PCA) score plot (Figure 3A) showed clear separation between the CON and LPR groups, as well as between the LPR and PCPH groups. Figure 3B,C, respectively, displayed the differentially expressed genes (DEGs) between CON vs. LPR and LPR vs. PCPH. Compared to the CON group, the LPR group had 1576 upregulated genes and 544 downregulated genes (Figure 3B). In contrast to the LPR group, the PCPH group had 278 upregulated genes and 1157 downregulated genes (Figure 3C). By comparing the two transcriptome datasets, 107 overlapping DEGs were identified (Figure 3D). Hierarchical clustering analysis of DEGs in Figure 3E revealed that these genes exhibited expression changes in the LPR group of mice, which were subsequently reversed by PCPH treatment. Further Gene Ontology (GO) analysis of the DEGs, as shown in Figure 3F, revealed that the biological process primarily focused on key biological pathways such as cellular process, biological regulation, and response to stimulus. In terms of cellular components, these processes mainly occurred in critical cellular structures such as cellular anatomical entity and intracellular compartments. Molecular function analysis highlighted the importance of protein binding, transcription regulator activity, and transporter activity. Kyoto Encyclopedia of Genes and Genomes (KEGG) pathway analysis (Figure 3G) demonstrated significant enrichment of DEGs in pathways such as NF-κB signaling pathway, PI3K/Akt signaling pathway, and TNF signaling pathway.

### 2.4. Metabolome Analysis Results

**Figure 4:**
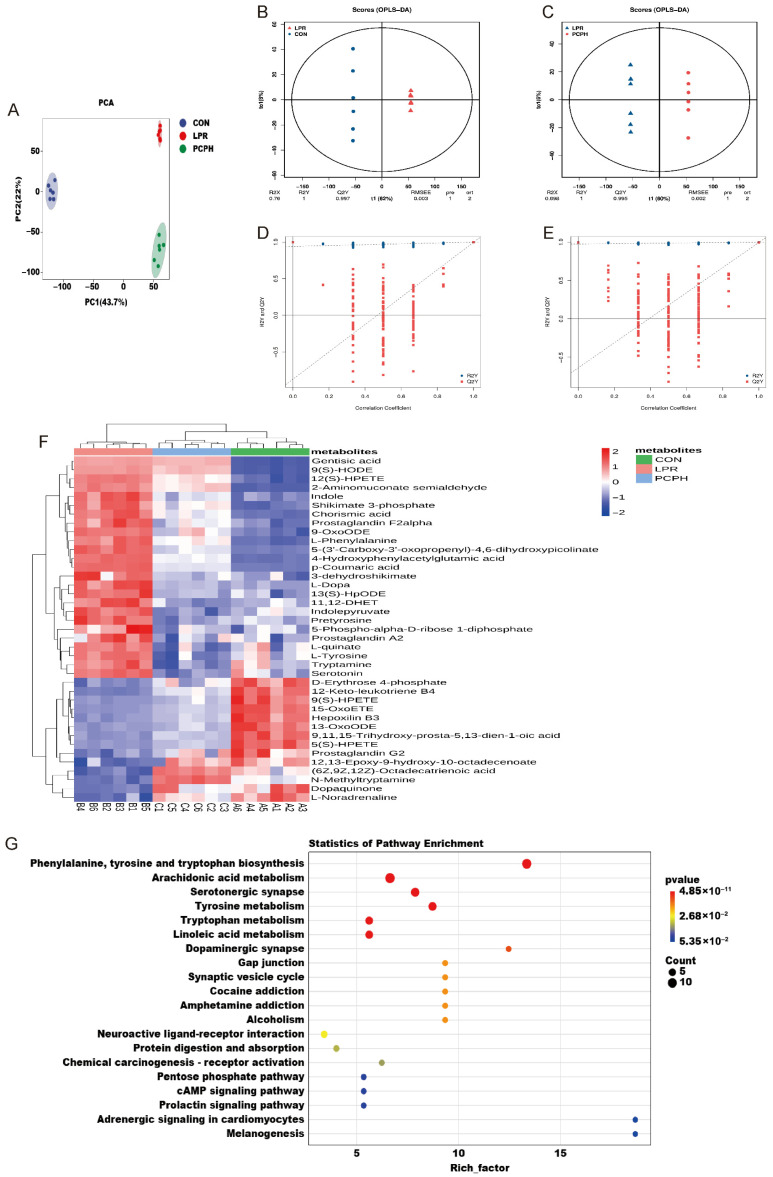
Metabolomics analysis results (*n* = 6). (**A**) PCA. (**B**) OPLS-DA analysis between the CON and LPR groups. (**C**) OPLS-DA analysis between the LPR and PCPH groups. (**D**) RPT analysis between the CON and LPR groups. (**E**) RPT analysis between the LPR and PCPH groups. (**F**) Heatmap of significantly differential metabolites among the CON, LPR, and PCPH groups. (**G**) KEGG metabolic pathway enrichment analysis. The color of the dots represents the *p*-value, with deeper red indicating more significant enrichment. The size of the dots represents the number of differentially enriched metabolites.

As shown in Figure 4A, the PCA score plot revealed tight clustering of samples within groups, indicating stable analytical conditions and good reproducibility of the detection process. Additionally, there was clear separation among the serum metabolites of the CON, LPR, and PCPH groups. The Orthogonal partial least squares discriminant analysis (OPLS-DA) model distinguished between the CON and LPR groups (Figure 4B), with cumulative R2Y and Q2Y values of 1 and 0.997, respectively, demonstrating the stability and reliability of the model. The cumulative R2Y and Q2Y values for the LPR and PCPH groups (Figure 4C) were 1 and 0.995, respectively, indicating the efficacy of PCPH treatment. Furthermore, the accuracy of the model was evaluated using the response permutation testing (RPT) method. As shown in Figure 4D,E, the OPLS-DA model exhibited good precision.

Based on the criteria of |log_2_ (fold change, FC)| > 0, Variable Importance in Projection (VIP) > 1, and *p* < 0.01, screening of the CON, LPR, and PCPH groups identified 38 candidate serum metabolites as potential biomarkers, primarily related to amino acid metabolism and lipid metabolism. Following PCPH administration, all potential biomarkers tended to revert to the levels observed in the CON group. The heatmap analysis (Figure 4F) demonstrated the significant impact of PCPH on the metabolic characteristics of MRL/lpr mice. Using the Biocloud platform pathways, including phenylalanine, tyrosine, and tryptophan biosynthesis, arachidonic acid metabolism, tyrosine metabolism, tryptophan metabolism, and linoleic acid metabolism, which were significantly enriched.

### 2.5. Integration of Metabolomics and Transcriptomics Analyses

**Figure 5:**
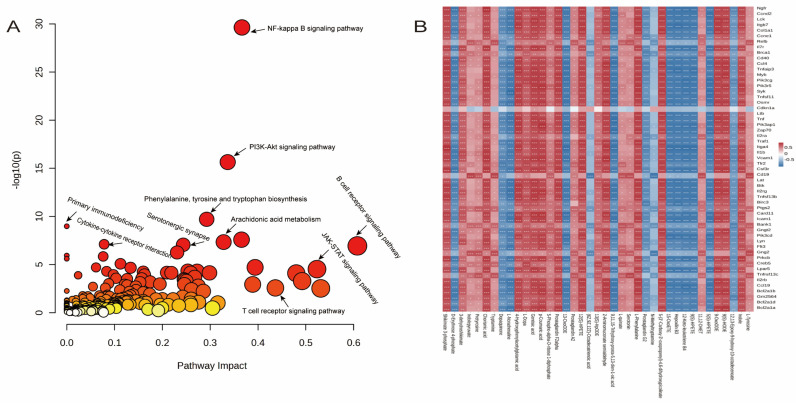
Integrated multi-omics analysis of PCPH in the treatment of LN. (**A**) Correlation analysis between common genes and potential biomarkers. (**B**) Joint pathway analysis of transcriptomics and metabolomics. Different colors represent the values of the correlation coefficients, with red indicating positive correlation and blue indicating negative correlation. * *p* < 0.05, ** *p* < 0.01, *** *p* < 0.001.

To gain a systems-level understanding of the therapeutic mechanism of PCPH on LN, an integrated analysis of transcriptomic and metabolomic data was performed. Lists of significant DEGs and differentially abundant metabolites (DAMs) were prepared, with gene identifiers converted to official gene symbols and metabolite identifiers mapped to KEGG compound IDs to ensure consistency. Subsequently, a joint pathway analysis was conducted using the ‘Joint Pathway Analysis’ module in MetaboAnalyst 6.0, which combines KEGG enrichment results from both omics layers and employs a Fisher’s exact test to evaluate the combined significance of pathway perturbations. To further explore the direct interactions between genes and metabolites, a correlation network was constructed by calculating pairwise Spearman correlation coefficients between all significant DEGs and DAMs. Only robust correlations (|ρ| > 0.5) with statistical significance (*p* < 0.05) were retained and visualized (as shown in Figure 5B). The results of the joint pathway analysis (Figure 5A) revealed that several key pathways, including the NF-κB signaling pathway, PI3K/Akt signaling pathway, and arachidonic acid metabolism, were significantly enriched (*p* < 0.05) when evidence from both transcripts and metabolites was considered together.

### 2.6. PCP Inhibits Key Proteins in the PI3K/AKT/NF-κB Pathway to Improve LN

**Figure 6:**
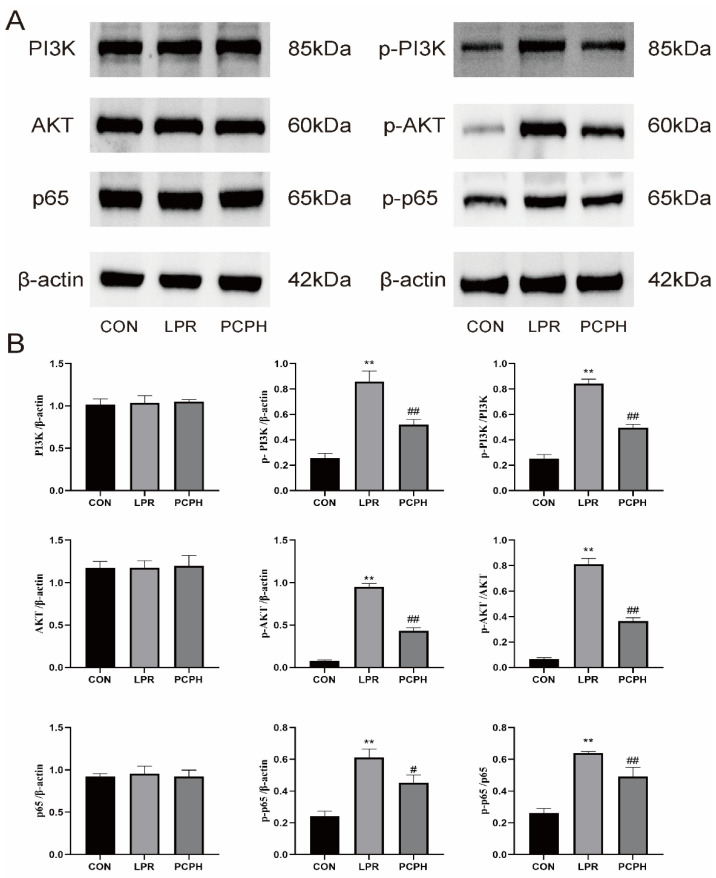
Western blot analysis of the expression of PI3K/AKT/NF-κB signaling pathway-related proteins. (**A**) Individual protein bands and groupings. (**B**) Relative protein expression levels of PI3K, p-PI3K, the ratio of p-PI3K to PI3K, AKT, p-AKT, the ratio of p-AKT to AKT, NF-кB (p65), p-NF-кB (p65), the ratio of p-NF-кB (p65) to NF-кB (p65). Data are presented as mean ± standard error (*n* = 3). Compared to the CON group, ** *p* < 0.01; compared to the LPR group, ^#^ *p* < 0.05, ^##^ *p* < 0.01.

Western blot analysis results indicated that the levels of p-PI3K, p-AKT, and p-p65 proteins, as well as the ratios of p-PI3K/total PI3K, p-AKT/total AKT, and p-p65/total p65, were significantly higher in the LPR group compared to the CON group (*p* < 0.01). In contrast, PCPH treatment effectively reduced the expression levels of p-PI3K (*p* < 0.01), p-AKT (*p* < 0.01), and p-p65 (*p* < 0.05) proteins, as well as the phosphorylation-to-total protein ratios (*p* < 0.01) (Figure 6A,B).

## 3. Discussion

SLE is an autoimmune disease that occurs in genetically susceptible individuals and is triggered by environmental factors. It is characterized by a loss of immune tolerance to nuclear and cytoplasmic autoantigens [17]. The pathogenesis involves abnormal activation of the innate immune system, which triggers a series of immune responses, including the activation of autoreactive B cells, the production of various autoantibodies, and the deposition of immune complexes in multiple organs [18]. These processes lead to tissue inflammation and damage, with LN being a typical and severe complication [19]. Therefore, exploring new treatment strategies and developing novel drugs are of great significance for improving the clinical therapeutic outcomes of LN.

With ongoing exploration of fungal components, the potential of PCP in treating LN has been identified. This study systematically investigated the effects of PCP on MRL/lpr mice from multiple aspects, including proteinuria, autoantibodies, inflammatory cytokines, and renal function indicators. The impact on the kidneys was observed using Enzyme-linked immunosorbent assays (ELISA), H&E staining, and Masson’s trichrome staining. Additionally, mechanisms of PCPH intervention in MRL/lpr mice were analyzed through transcriptomics, Ultra-high performance liquid chromatography-MS based untargeted metabolomics, Western blot, and other techniques.

In this study, the MRL/lpr mouse model, one of the most used animal models in LN research, was utilized. These mice are genetically predisposed to SLE due to a mutation in the gene encoding the Fas protein, leading to spontaneous production of autoantibodies and the development of lupus-like symptoms [20]. Administration of both PCPL and PCPH to MRL/lpr mice effectively restored proteinuria levels. Additionally, elevated levels of autoantibodies (anti-dsDNA and ANA), which are biomarkers for LN that increase during its progression, were observed in the MRL/lpr mice [21]. Concurrently, levels of pro-inflammatory cytokines (TNF-α and IL-6), which are used for the diagnosis and monitoring of LN, were also elevated. BUN and Cr are indicators used to assess renal function status [22]. However, In this study, intervention with either PCPL or PCPH led to significant improvements in MRL/lpr mice, characterized by reduced levels of autoantibodies and inflammatory cytokines, as well as enhanced renal function. Histological analysis confirmed these systemic benefits; both H & E and Masson’s trichrome staining revealed that the treatments markedly ameliorated renal tubular degeneration and reduced fibrosis, thereby restoring kidney structure and function. Notably, a comparative analysis of these outcomes indicated that PCPH provided superior renal protection. This pronounced efficacy prompted us to focus subsequent transcriptomic and metabolomic investigations on PCPH to elucidate its underlying therapeutic mechanisms.

Transcriptomic analysis revealed that the DEGs among the three groups were significantly enriched in the NF-κB signaling pathway and the PI3K/Akt signaling pathway. This suggests that PCPH may exert its multi-target therapeutic effects through these pathways and highlights the importance of the PI3K/AKT/NF-κB pathway as a key pathological feature in LN. NF-κB, a core regulator of immune inflammation, is excessively activated. This is characterized by enhanced nuclear translocation of its p65 subunit in the glomerular mesangial area, which promotes the overexpression of pro-inflammatory cytokines (such as TNF-α and IL-6) and chemokines. This process accelerates inflammatory damage in both the glomeruli and renal tubules. Additionally, NF-κB upregulates complement components, leading to immune complex deposition and activation of the complement cascade, which causes damage to the glomerular basement membrane and apoptosis of tubular epithelial cells [23]. Concurrently, the aberrantly activated PI3K/Akt pathway not only promotes B-cell survival, antibody class switching, and the formation of immune complexes—all of which contribute to renal injury—but also stimulates the proliferation of glomerular mesangial cells and the deposition of extracellular matrix, thereby accelerating glomerulosclerosis. More importantly, the PI3K/Akt pathway can phosphorylate and inhibit the NF-κB inhibitory factor IκBα, which in turn promotes the nuclear translocation of NF-κB. Thus, PI3K/Akt and NF-κB form a dual inflammatory signal amplification loop that synergistically exacerbates renal inflammation and tissue damage in LN [24].

Metabolomic analysis has identified 38 potential biomarkers closely associated with LN disruption, which were significantly enriched in phenylalanine, tyrosine and tryptophan biosynthesis, arachidonic acid metabolism, tyrosine metabolism, tryptophan metabolism, and linoleic acid metabolism. This suggests that PCPH may intervene in LN-related inflammatory responses and immune regulation by modulating key enzymes or metabolites in these metabolic pathways. In LN, damage to the glomerular filtration barrier and renal tubules impairs amino acid reabsorption, leading to an imbalance in plasma levels of branched-chain amino acids (BCAAs) and aromatic amino acids. Specifically, BCAAs (leucine, isoleucine, valine) are abnormally elevated in the blood of LN patients [25]. Their metabolites activate the NF-κB pathway, inducing the release of pro-inflammatory cytokines (TNF-α, IL-6) and secretion of matrix metalloproteinases, which accelerate glomerular basement membrane degradation and renal tubulointerstitial fibrosis [26]. Notably, the PI3K/AKT/NF-κB signaling pathway serves as both a core regulator of amino acid metabolism and a central hub for inflammation and immune injury. PI3K/AKT promotes NF-κB nuclear translocation through phosphorylation of IκBα, forming a dual signal amplification loop that drives abnormal B-cell activation, immune complex deposition, and mesangial cell proliferation [27]. Based on this, PCPH may target the PI3K/AKT/NF-κB pathway to regulate amino acid metabolic disorders and interrupt the vicious cycle of inflammation and metabolism, thereby exerting multi-dimensional therapeutic effects on LN.

Further integrated transcriptomic and metabolomic analysis revealed significant enrichment of the NF-κB signaling pathway, PI3K/Akt signaling pathway, JAK/STAT signaling pathway, phenylalanine, tyrosine and tryptophan biosynthesis, and arachidonic acid metabolism during the treatment of LN with PCPH. Notably, the PI3K/AKT/NF-κB signaling pathway plays a critical role in immunometabolism, inflammation, and immune responses. It is directly associated with inflammation, immune imbalance, and renal fibrosis, and promotes the proliferation and activation of renal fibroblasts—key factors in kidney injury. Therefore, subsequent research will focus on investigating the specific mechanisms of PCPH in modulating the PI3K/AKT/NF-κB signaling pathway during the treatment of LN.

P-PI3K drives abnormal B-cell activation and autoantibody production, leading to the deposition of immune complexes in the kidneys. As an upstream activator of the AKT pathways, it directly promotes renal inflammation and fibrosis. Clinical evidence shows that the expression of P-PI3K in the renal tissues of LN patients is significantly higher than in healthy controls and is positively correlated with disease activity [28]. AKT is a key kinase downstream of PI3K. Its phosphorylated form, P-AKT, is fully activated through dual-site phosphorylation at Thr308 and Ser473, regulating cell proliferation, survival, and metabolism [10]. In LN, P-AKT upregulates NF-κB P65 activity, promoting the release of inflammatory cytokines such as TNF-α and IL-6, thereby amplifying local inflammation. Clinical studies have confirmed elevated levels of P-AKT in the serum and renal tissues of LN patients, which positively correlate with renal function indicators such as proteinuria and Cr [29]. The phosphorylated form of P65, P-P65, enters the nucleus and initiates the transcription of pro-inflammatory genes. In LN, P-P65 upregulates the expression of factors such as TNF-α, and IL-6, recruiting macrophages to infiltrate the kidneys and exacerbating renal tubulointerstitial inflammation. Through its interaction with the PI3K/AKT pathway (where P-AKT directly phosphorylates P65), it forms a positive feedback loop that amplifies inflammatory signals. Additionally, it induces fibrosis factors like TGF-β, accelerating renal tissue fibrosis [30]. Results demonstrate that PCPH treatment significantly reduces the expression levels of key signaling pathway proteins, including p-PI3K, p-AKT, and p-p65, as well as the phosphorylation levels of these proteins relative to their total protein expression.

In conclusion, this study systematically elucidated that PCP achieves effective treatment of LN in the MRL/lpr mouse model through the precise modulation of the PI3K/AKT/NF-κB signaling pathway, providing solid experimental evidence for its potential as a therapeutic strategy for LN. However, we must also candidly acknowledge a limitation of our study: our conclusions rely heavily on a single animal model, the MRL/lpr. Although this model is widely used in LN research due to its rapid and severe disease progression, LN exhibits significant heterogeneity across different genetic backgrounds. We plan to conduct subsequent experiments in other classic LN models, such as NZB/W F1. By confirming its therapeutic efficacy across multiple preclinical models, we aim to construct a more comprehensive pharmacodynamic profile for PCP, thereby laying a more solid foundation for its subsequent application.

## 4. Materials and Methods

### 4.1. Preparation of PCP

For the cultivation of *Irpex lacteus* and the extraction of its polysaccharides, the following experimental procedures were implemented. First, the slant culture of *Irpex lacteus* was activated at room temperature for 4 h, and then aseptically transferred to a liquid shake-flask medium under sterile conditions. The composition of this medium included 3% glucose, 2% yeast extract, 0.2% potassium dihydrogen phosphate, 0.15% magnesium sulfate, and 10 mg of vitamin B_1_, all dissolved in 1000 mL of distilled water. The prepared medium was then dispensed, sealed, and autoclaved at 121 °C for 30 min before use.

Next, the activated *Irpex lacteus* slant culture was aseptically transferred into the prepared liquid shake-flask medium. The flasks were placed in a constant temperature orbital shaker incubator and cultivated at 28 °C and 180 r·min^−1^ for 5 days to obtain a liquid seed culture. A stable liquid seed culture for subsequent experiments was obtained through subculturing for 3–4 days with each passage.

Subsequently, the liquid seed culture was inoculated into a 5 L fermenter. The medium formulation for the fermenter was as follows: 1.5% starch, 1.5% glucose, 3% wheat bran, 3–5% soybean cake, 1% peptone, 0.2% potassium dihydrogen phosphate, 0.15% magnesium sulfate, and 0.2% calcium chloride. The wheat bran and soybean cake were boiled with an equal volume of water for approximately 15 min, filtered, and then added to the fermented medium. The fermenter was autoclaved at 121 °C for 60 min. It was then placed on a magnetic stirrer equipped with a cooling system. Once the temperature dropped to 28 °C, the liquid seed culture was inoculated, and cultivation continued for 180 h.

After fermentation was complete, the 5 L fermenter was disconnected, and the mycelia were separated by filtration. The mycelial biomass was then mixed with a certain volume of water and extracted twice, for one hour each time. The mycelial extracts and the fermentation broth were combined, concentrated, and then cooled. Ethanol was added to a final concentration of approximately 80%, and the mixture was left to stand at 4 °C for 48 h. Subsequently, the mixture was vacuum filtered to collect the precipitate. The precipitate was air-dried to obtain the ethanol-precipitated fraction of *Irpex lacteus*.

Finally, the ethanol-precipitated fraction was dissolved in deionized water and deproteinized using Sevag reagent (a 4:1 *v*/*v* mixture of chloroform: n-butanol). The mixture was centrifuged to remove the precipitate, and the supernatant was recovered. The supernatant was placed in a dialysis bag and soaked in flowing water for 48 h to remove small molecules. The dialyzed polysaccharide solution was then freeze-dried for the subsequent analysis of PCP.

### 4.2. Physical and Chemical Analyses of PCP

The total PCP content was evaluated using the well-established phenol-sulfuric acid method, with glucose serving as the standard for comparison. The soluble glycoprotein content was quantified through Coomassie brilliant blue staining. Sulfate content was measured using barium chloride gelatin turbidimetry, employing bovine serum albumin and the sulfate radical as respective standards.

For UV absorption spectrometry, a 0.5 mg/mL solution of PCP in deionized water was scanned over the wavelength range of 190–400 nm using a UV-Vis spectrophotometer (manufactured by Shimadzu Experimental Equipment Co., Ltd., Shanghai, China) with a 1 nm scan interval. IR absorption spectroscopy was conducted using a Nicolet iZ-10 spectrometer (Thermo Fisher Scientific, Waltham, MA, USA). PCP samples were mixed with KBr powder and compressed into 1 mm pellets for Fourier-transform IR measurements spanning from 4000 to 400 cm^−1^. 

Monosaccharide compositions were determined using high-performance anion-exchange chromatography with a CarboPac PA-20 anion-exchange column (3 mm × 150 mm; Dionex, Thermo Fisher Scientific, Hamburg, Germany) and a pulsed amperometric detector (PAD; Dionex ICS 5000+ system, Thermo Fisher Scientific, Hamburg, Germany). The chromatographic conditions were as follows: flow rate, 0.5 mL/min; injection volume, 5 μL. The solvent system consisted of solvent A (ddH_2_O), solvent B (0.1 M NaOH), and solvent C (0.1 M NaOH, 0.2 M NaAc). The gradient program was set as follows: the volume ratio of solutions A, B, and C was 95:5:0 at 0 min, 85:5:10 at 26 min, 85:5:10 at 42 min, 60:0:40 at 42.1 min, 60:40:0 at 52 min, 95:5:0 at 52.1 min, and 95:5:0 at 60 min. Monosaccharide compositions were determined using high-performance anion-exchange chromatography with a CarboPac PA-20 anion-exchange column (3 mm × 150 mm; Dionex, today part of Thermo Fisher Scientific, Hamburg, Germany) and a pulsed amperometric detector (PAD; Dionex ICS 5000+ system, Thermo Fisher Scientific, Hamburg, Germany). 

### 4.3. Animals and Experimental Design

Eight-week-old BALB/c mice and MRL/lpr mice were purchased from SPF Biotechnology Co., Ltd. (Beijing, China), which holds the license number SCXK (Beijing, China) 2019-0010. All experimental procedures were strictly conducted in accordance with the guidelines of the Ethics Committee of Changchun University of Traditional Chinese Medicine, and the study was approved by the committee with the approval number 2023514. The mice were housed in a barrier environment and provided with standardized husbandry conditions and diet. After a one-week acclimatization period to the laboratory environment, the BALB/c mice were designated as the CON group. The MRL/lpr mice were then randomly divided into four groups, with six mice in each group. The groups included the LPR group, the PAT group (PAT, PAT, receiving 5 mg Prednisone acetate tablet/kg body weight), the PCPL group (PCPL, administered with 1 g/kg body weight of PCP), and the PCPH group (PCPH, administered with 2 g/kg body weight of PCP). During the intervention phase, the mice in the PCPL, PCPH and PAT groups were administered the corresponding doses of drugs via gavage, while the mice in the CON and LPR groups were given an equal volume of distilled water via gavage. The entire experimental period lasted for 8 weeks, during which urine samples were collected every two weeks for proteinuria detection, resulting in a total of five measurements. At the end of the experiment (week 8), all mice were euthanized. Subsequently, serum was separated by centrifugation at 3000 rpm for 10 min; simultaneously, one kidney was harvested and rapidly frozen in liquid nitrogen for storage, with all procedures strictly adhering to aseptic principles throughout.

### 4.4. ELISA

Proteinuria, anti-dsDNA, ANA, Sm, TNF-α, IL-6, BUN, Cr levels were measured following the manufacturer’s instructions provided with the kits (Nanjing Jiancheng Bioengineering Institute, Nanjing, China).

### 4.5. Histopathological Analysis of Kidney Tissue

Kidney tissue, initially fixed in 4% paraformaldehyde, underwent a series of dehydration steps using an alcohol gradient, followed by immersion in paraffin wax. The wax-embedded tissue was subsequently frozen and trimmed, and the cooled wax blocks were placed in a paraffin microtome. Sections with a thickness of 4 μm were carefully mounted onto glass slides. H & E staining and Masson’s trichrome staining were then applied to these sections. Following staining, the sections were scanned, allowing for detailed observation of the kidney tissue pathology.

### 4.6. Kidney Transcriptomics

Total RNA from mouse kidneys was extracted using TRIzol reagent from Life Technologies. Following extraction, the concentration and purity of the RNA were determined using NanoDrop 2000 (Thermo Fisher Scientific, Wilmington, DE, USA), while its integrity was assessed using an Agilent Bioanalyzer 2100 system (Agilent Technologies, Santa Clara, CA, USA). Starting with 1 μg of total RNA, sequencing libraries were prepared, including steps for mRNA enrichment, cDNA synthesis, end repair, adapter ligation, and PCR amplification, using a kit from Yeasen Biotechnology (Shanghai, China) Co., Ltd. The quality of the libraries was then evaluated on the Agilent Bioanalyzer 2100. The libraries were sequenced on the Illumina NovaSeq platform using 150 bp paired-end sequencing. Subsequent bioinformatics analysis was performed on the BMKCloud platform. A comprehensive and reproducible bioinformatics pipeline was developed in R version 4.3.1. All package versions were recorded using sessionInfo() for full transparency. Differential expression analysis was performed with the DESeq2 package (v1.44.0), utilizing dplyr (v1.1.4) and tibble (v3.2.1) for data manipulation. DEGs were identified based on a threshold of *p* < 0.01 and |log_2_FC| > 1. Key results were visualized using ggplot2 (v3.5.1) for volcano plots and pheatmap (v1.0.12) for hierarchical clustering heatmaps. Furthermore, functional enrichment analysis of GO and KEGG Ortholog terms was conducted using the clusterProfiler package (v4.12.3), with visualizations generated by enrichplot (v1.24.0). Pathway enrichment analysis was performed using hypergeometric distribution testing. The resulting raw *p*-values were adjusted for multiple testing using the Benjamini–Hochberg method to control the false discovery rate (FDR). A pathway with an adjusted *p*-value (FDR) < 0.05 was considered significantly enriched.

### 4.7. Serum Metabolomics

Metabolomics analysis was conducted using a Waters Acquity I-Class PLUS ultra-high-performance liquid chromatography system coupled with a Waters Xevo G2-XS Q-TOF high-resolution mass spectrometry system, employing a Waters Acquity UPLC HSS T3 column. The mass spectrometer simultaneously acquired MS and MS/MS data in both positive and negative ion modes under MSe mode, with a low collision energy of 2 V and a high collision energy ranging from 10 to 40 V. ESI ion source parameters were set according to the ion mode. Raw data acquired using MassLynx V4.2 underwent peak picking, alignment, and metabolite identification using Progenesis QI (integrating the METLIN online database and an in-house Biomark library, with theoretical fragment identification and mass deviation controlled within 100 ppm). Following normalization of peak areas, repeatability and quality were assessed using PCA and Spearman correlation analysis. Differences between groups were evaluated using *t*-tests to obtain *p*-values and by calculating |log_2_FC|. OPLS-DA was performed for modeling and validation using the ropls package (v1.38.0). Metabolites were considered significantly different if they met all three criteria: *p* < 0.01, a Variable Importance in VIP score > 1 from the OPLS-DA model, and |log_2_FC| > 0. Functional annotation of these metabolites, including compound classification and pathway information, was retrieved from the KEGG, Human Metabolome Database (HMDB), and LIPID MAPS databases. Pathway enrichment analysis was conducted to identify significantly altered pathways. This analysis utilized a hypergeometric distribution test to determine whether the number of DAMs within a given pathway was greater than expected by chance. The test was performed against a background set of all expressed metabolites identified in our study. The resulting *p*-values were adjusted for multiple hypothesis testing using the Benjamini–Hochberg FDR method, and pathways with an adjusted *p*-value (FDR) < 0.05 were considered significantly enriched. All statistical analyses and data visualizations were conducted in R (v4.3.2), primarily using packages from the tidyverse (v2.0.0), including dplyr (v1.1.4) for data manipulation and ggplot2 (v3.5.1) for plotting.

### 4.8. Western Blot Analysis

The expression levels of p-PI3K, PI3K, p-AKT, AKT, NF-κB p65, and NF-κB p-p65 in mouse kidney tissues were detected using Western blotting. Primary antibodies including PI3K (AF6241), AKT (60203-1-IG), p65 (28273-1-AP), and β-actin (60004-1-IG) were purchased from Wuhan Sanying Biotechnology Co., Ltd. (Wuhan, China). Antibodies for p-PI3K (LJS-A-3241), p-AKT (66444-1-IG), and p-p65 (582335-1-RR) were obtained from Cell Signaling Technology, Inc. (Danvers, MA, USA).

The minced renal tissue was homogenized in radioimmunoprecipitation assay (RIPA) lysis buffer to denature proteins. The homogenate was then vigorously agitated and centrifuged at 12,000 rpm and 4 °C for 10 min to collect the supernatant. The protein concentration of the supernatant was quantified using the bicinchoninic acid assay (BCA) method. After cooling, the samples were stored at −20 °C. The prepared samples were separated by electrophoresis, transferred onto a membrane, and blocked. The membrane was subsequently incubated overnight at 4 °C with primary antibodies specifically targeting PI3K, p-PI3K (1:1000 dilution), AKT, p-AKT (1:2000 dilution), p-p65, p65 (1:3000 dilution), and β-actin (1:10,000 dilution). Following primary antibody incubation, the membrane was treated with a horseradish peroxidase (HRP)-conjugated secondary antibody (1:10,000 dilution) for 2 h at room temperature. After washing, the protein bands were visualized using an enhanced chemiluminescence (ECL) reagent and a chemiluminescence imaging system (SH-523; Hangzhou Shenhua Technology Co., Ltd., Hangzhou, China). Finally, the images were captured and analyzed using ImageJ software (version 1.46). 

### 4.9. Statistical Analysis

Data are presented as the mean ± standard error of the mean. Statistical analysis was conducted with GraphPad Prism 9.5 software (GraphPad Software, Inc., Boston, MA, USA). To assess differences between groups, a one-way analysis of variance was employed, and the results were visualized using charts. A *p*-value of less than 0.05 was considered to indicate statistical significance.

## 5. Conclusions

This study found that PCP demonstrates remarkable pharmacodynamic effects in MRL/lpr mice. Specifically, PCPH effectively alleviated LN symptoms in MRL/lpr mice by inhibiting the PI3K/AKT/NF-κB signaling pathway. These results provide strong theoretical support for the clinical application and potential development of PCP-based new drugs.

## Figures and Tables

**Table 1:** Chemical components of PCP.

Physicochemical Indexes	Standard Curve	Correlation Coefficient	Content (mg/g)
Polysaccharide	Y= 379.44x − 26.778	0.9995	696.86 ± 6.54
Soluble protein	Y = 1.1688x + 0.0269	0.9991	11.51 ± 0.08
Sulfate radical	Y = 2.2451x − 0.0146	0.9988	94.40 ± 1.36

## Data Availability

The original contributions presented in this study are included in the article. Further inquiries can be directed to the corresponding author(s).

## Abbreviations

The following abbreviations are used in this manuscript:

Anti-dsDNA	Anti-double-stranded DNA antibodies
ANA	Anti-nuclear antibodies
Anti-Sm	Anti-Sm antibody
Ara	Arabinose
BUN	Blood urea nitrogen
BCAAs	Branched-chain amino acids
BCA	Bicinchoninic acid assay
Cr	Creatinine
CON	Control
DEGs	Differentially expressed genes
DAMs	Differentially abundant metabolites
ELISA	Enzyme-Linked Immunosorbent Assay
ECL	Enhanced chemiluminescence
Fuc	Fucose
Fru	Fructose
FDR	False discovery rate
FC	Fold Change
Gal	Galactose
Glc	Glucose
Gal-UA	Galacturonic acid
Glc-UA	Glucuronic acid
Gul-UA	Guluronic acid
GO	Gene Ontology
HMDB	Human metabolome database
H&E	Hematoxylin and Eosin
HRP	Horseradish peroxidase
IR	Infrared
IL-6	Interleukin-6
KEGG	Kyoto Encyclopedia of Genes and Genomes
LN	Lupus nephritis
LPR	Model
Man	Mannose
Man-UA	Mannuronic acid
OPLS-DA	Orthogonal partial least squares discriminant analysis
PCP	*Irpex lacteus* polysaccharides
PAT	Prednisone acetate tablet
PCPH	High-dose PCP
PCPL	Low-dose PCP
PCA	Principal component analysis
Rha	Rhamnose
Rib	Ribose
RPT	Response permutation testing
RIPA	Radioimmunoprecipitation assay
SLE	Systemic lupus erythematosus
TNF-α	Tumor necrosis factor-alpha
UV	Ultraviolet
VIP	Variable Importance in Projection
Xyl	Xylose
